# Comparison of diagnostic tools to assess the feasibility of programmatic use of rapid diagnostic tests for onchocerciasis: A dataset from Gabon

**DOI:** 10.1016/j.dib.2024.110901

**Published:** 2024-09-06

**Authors:** Julienne Atsame, Jacob N. Stapley, Aditya Ramani, Romain Mourou, Ella Ntsame, Eya Efame, Ollomo-Nziengui Angue, Jean-Luc Obiang, Nils Pilotte, Katherine Gass, Maria-Gloria Basáñez

**Affiliations:** a Programme de Lutte contre les Maladies Parasitaires, Ministère de la Santé. Libreville, Gabon; b MRC Centre for Global Infectious Disease Analysis and London Centre for Neglected Tropical Disease Research, School of Public Health, Imperial College London, London, UK; c Department of Pathobiology and Population Sciences, Royal Veterinary College, Hatfield, UK; d Département de Parasitologie et de Mycologie. Université des Sciences de la Santé. Libreville, Gabon; e Laboratoire Professeur Gahouma Daniel. Libreville, Gabon; f Department of Biological Sciences, Quinnipiac University, Hamden, CT, USA; g Task Force for Global Health, Decatur, Georgia, USA

**Keywords:** Onchocerciasis, Hypoendemicity, Delineation mapping, Microfilariae, Ov16 RDT, Ov16 ELISA

## Abstract

Due to the success of large-scale ivermectin mass drug administration (MDA), the aim of onchocerciasis intervention efforts have shifted from control of the disease to elimination of transmission. This has necessitated a greater understanding and comparison of the performance of diagnostic tools in hypoendemic (low prevalence) settings which had not been incorporated into large-scale MDA programmes before the goal switched from onchocerciasis elimination as a public health problem to elimination (interruption) of transmission (EOT). Data on age, sex and duration of residence were collected, prior to ivermectin treatment, across Gabon in 2015 from 5,829 participants in 67 communities from 14 districts. Skin-snip samples (for detection of *Onchocerca volvulus* microfilariae) were obtained from 4,350 (75 %) and blood samples (for detection of presence of IgG4 antibodies against the *O. volvulus* Ov16 antigen) from 4,257 of those skin-snip tested (98 %).

Whole blood was tested in the field using the SD Ov16 Rapid Diagnostic Test Prototype (Ov16 RDT). Dried blood spots (DBS) were prepared for all blood-sampled individuals. After assessing DBS quality, 2,990 (70 %) samples underwent valid analysis in the lab using horseradish peroxidase (HRP) Ov16 enzyme-linked immunosorbent assay (Ov16 ELISA). The number of positive individuals varied between diagnostic tools with skin-snip microscopy, Ov16 RDT and Ov16 ELISA detecting 337/4,350 (8 %, 95 % CI =7 %–9 %), 383/4,257 (9 %, 8 %–10 %) and 348/2,990 (12 %, 10 %–13 %), respectively. Data were analysed to understand the age profiles of microfilarial and IgG4 antibody prevalence by diagnostic and mapped across Gabon.

These data have reuse potential for policy makers, test manufacturers and country programmes when making determinations at community level of the suitability of using Ov16 RDT for conducting delineation mapping or evaluating the current stage of a community or, more generally, an evaluation unit along the EOT path. Further, these data are of use to transmission dynamics modellers who can fit models to these data to better understand the stage(s) in the *O. volvulus* lifecycle likely responsible for IgG4 antibody seroconversion in the presence of Ov16 antigen. This is crucial for incorporation of antibody prevalence as an output of onchocerciasis transmission models to permit evaluation of currently proposed serological thresholds to inform decisions about Start- or Stop-MDA in the context of onchocerciasis elimination mapping (OEM) and verification of EOT, respectively.

Specifications Table

**Table:** 

Subject	Health and Medical Sciences – Infectious Diseases
Specific subject area	Evaluation of diagnostic tools for mapping and surveillance within regions hypoendemic for onchocerciasis
Type of data	.xlsx files: Stapley, Jacob N (2024), “Dataset of the performance of Ov16 RDTs versus skin snip microscopy and Ov16 ELISA, Gabon, 2015″, Mendeley Data, V2, doi: 10.17632/vtvmrzs9ch.2 [1] Tables: Table 1 Graphs: Fig. 2 Diagrams: Fig.s 1 & 3 Raw: .xlsx file Analysed: Table 1; Fig.s 1–3
Data collection	Skin-snip microscopy: Bloodless skin-snip samples were collected from the right and left iliac crests using a 2-mm Holth corneoscleral punch. Following 24-hour incubation of the snips in saline solution in a microtitration plate's wells, the medium was spread onto microscope slides and examined under light microscopy to detect *O. volvulus* microfilariae in the skin.
	IgG4 antibodies against the *O. volvulus* Ov16 antigen: Finger-prick blood samples were collected; 15 µL for the SD RDT Prototype (Standard Diagnostics, Inc.) and 100 µL dried onto Whatman 903 filter paper. Dried blood spots (DBS) were eluted in phosphate buffer saline tween (PBST) in the lab, with the eluate used in an HRP Ov16 ELISA.
Data source location	*Country:* Gabon (nationwide)*.*
Data accessibility	Repository name: Mendeley Data Data identification number: doi:10.17632/vtvmrzs9ch.2 Direct URL to data: https://data.mendeley.com/datasets/vtvmrzs9ch/2
Related research article	none*.*

## 1 Value of the data

• Why are these data useful?
  Having data for skin-snip microscopy, Ov16 RDT and Ov16 ELISA for the same individuals and across all ages in treatment-naïve settings allows for comparison among tests and evaluation of RDT performance, crucial for assessing its programmatic usefulness, particularly in delineation mapping of areas yet to start MDA.
• Who can benefit from these data?
  Beneficiaries of these data include: a) policy makers involved in the development and review of guidelines for OEM [2]; b) test manufacturers gaining further understanding of their diagnostic tools' functionality in field epidemiological settings; c) country programmes assessing the usability and acceptability of less invasive testing procedures to increase participation rates by endemic communities in onchocerciasis surveillance activities [3,4].
• Programmatic applications:
  Diagnostic performance of Ov16 RDT could be used to assess whether RDT can replace both skin-snip microscopy and ELISA in the assessment of onchocerciasis prevalence and in turn be suitable for both OEM and Stop-MDA decisions on the path to verification of EOT.
• Transmission modelling applications:
  Testing various hypotheses on which *O. volvulus* lifecycle stage(s) is/are responsible for IgG4 seroconversion in response to Ov16 in humans and occurrence of seroreversion. Integrating and validating IgG4 antibody prevalence model outputs [[5], [6], [7], [8]]. Simulating IgG4 antibody prevalence age profiles under different pre- and post-MDA epidemiological scenarios to examine the suitability of proposed age groups and serological thresholds to inform Start-MDA and Stop-MDA decisions.

## 2 Background

Onchocerciasis interventions initially aimed to control *Onchocerca volvulus* infection to the extent it would no longer be a public health problem, focusing on regions with high levels of transmission and morbidity arising from ocular and skin disease. Given the success of ivermectin mass drug administration (MDA) programmes, the paradigm shifted from disease control to elimination (interruption) of transmission (EOT) [9]. In endemic countries, nationwide EOT requires that interruption of transmission be reached in all foci, including those previously non-prioritised because of their low baseline prevalence. This necessitates mapping and assessing hypoendemic areas for their integration into ivermectin treatment programmes (onchocerciasis elimination mapping, OEM). Parasitological diagnosis relies on skin-snip microscopy, a mildly painful and relatively invasive procedure which has low sensitivity in low-prevalence areas [10]. Tests that use finger-prick blood sampling to detect IgG4 antibodies to the *O. volvulus* Ov16 antigen represent a less invasive option. Conducting ELISA tests from these blood samples can be time-consuming and resource-intensive in endemic settings, with point-of-care rapid diagnostic tests (RDTs) offering a more practical alternative. We describe a dataset from delineation mapping in Gabon that includes skin-snip, Ov16 RDT and Ov16 ELISA data from the same individuals in hypoendemic communities prior to ivermectin treatment.

## 3 Data Description

All data were gathered through the analysis of skin-snip microscopy and blood samples collected for onchocerciasis delineation mapping across Gabon as part of a multi-country study to compare diagnostic tools, endorsed by the African Programme for Onchocerciasis Control (APOC) [11,12]. The raw data were entered into an .xlsx. file [1].

## 4 Experimental Design, Materials and Methods

### 4.1 Experimental design

Study sites were selected to conduct onchocerciasis delineation mapping in Gabon. A previous mapping exercise, based on prevalence of palpable nodules (where a proportion of adult *O. volvulus* worms reside) termed REMO (rapid epidemiological mapping of onchocerciasis), had determined that most districts in Gabon were hypoendemic (low prevalence) [15]. Another mapping exercise, based on the history of eye-worm (*Loa loa*) had also determined that most of the country was co-endemic with loiasis [16]. Therefore, Gabon had not received ivermectin treatment.

Biological samples were collected from community members who consented to participate, with the sampling frame determined by the total number of communities selected by APOC for OEM during 2015 [11].

In each of the communities, convenience sampling was conducted to select a total of up to 300 individuals. Individuals aged 5 years and older, who consented themselves to be examined (or via their parents/guardians) were included, with blood samples only taken from those who agreed to be skin snipped. Sample collectors used a census form which listed the identification (ID) numbers assigned to each consenting participant.

### 4.2 Skin-snipping

Both right and left iliac crests of the participants (*n* = 4350) were cleaned using an alcohol-based swab. Two bloodless skin-snip samples were collected from the crests using a 2-mm Holth corneoscleral punch (Holth, New York, NY, USA) with each biopsy placed into a well of a microtitration plate. Following 24-hour incubation of the snips in saline solution, the medium, into which microfilariae emerged, was spread onto microscope slides and examined under (low magnification) light microscopy to detect the presence of and enumerate *O. volvulus* microfilariae in the skin snips [17]. All Holth punches were sterilised after every use to maintain aseptic conditions.

### 4.3 Blood sampling

#### 4.3.1 Detection of IgG4 antibodies to Ov16 antigen using rapid diagnostic test (Ov16 RDT)

Blood samples were collected (*n* = 4257) from consenting participants (who had been skin-snipped) by finger-prick and 15 µL were used for Ov16 RDT. The test applied was the SD Ov16 Rapid Diagnostic Test Prototype (Standard Diagnostics, Inc., Suwon city, Republic of Korea). This is an immuno-chromatographic strip test that detects human IgG4 antibodies to the *O. volvulus* Ov16 antigen present in the blood sample. The test strip is composed of an absorbent pad, a nitrocellulose membrane striped with a control line reagent, a test reagent and a conjugate pad which contains dried colloidal gold detector reagent. The test line is a dried preparation of a recombinant fusion protein of glutathione-S-transferase and the Ov16 protein from *O. volvulus*, produced at PATH (Program for Appropriate Technology in Health, Seattle, WA, USA). The control line is a dried preparation of a commercially available goat anti-mouse IgG antibody. The detection reagent is a mouse anti-human IgG4 and colloidal gold nanoparticle conjugate, dried onto a glass-fibre conjugate pad. The sample and rehydrated detection reagent flow up the strip via capillary action through the nitrocellulose membrane when Ov16 buffer is added to the test. The immunoglobulins in the sample may bind the Ov16 antigen line in the nitrocellulose and the detection reagent may subsequently bind to the human immunoglobulins and will also bind to the control line antibody in the nitrocellulose. Results are visually interpreted at 20 min. If the sample contains IgG4 antibodies to the *O. volvulus* Ov16 antigen, the complex binds to the antigen in the test line, producing a red line indicating a positive result. In the absence of IgG4 antibodies to *O. volvulus* Ov16, no red line will form in the test line area, indicating a negative result. Absence of the control line indicates an invalid result [18].

#### 4.3.2 Preparation of dried blood spots

Blood samples (*n* = 4257) were collected by finger prick (100 µL) and placed onto Whatman 903 filter paper to prepare dried blood spots (DBS). Four aliquots of 6 drops of blood (25 µL for each aliquot, for approximately 4 µL per drop) were collected from each individual and absorbed onto the filter paper. Blood spots were labelled with their corresponding ID, dried, individually wrapped and stored at 4 °C, and subsequently at −20 °C until shipped to the testing laboratory. Only DBS of suitable quality, determined by examining the front and the back of the filter paper to ensure complete saturation (*n* = 3012), were tested for Ov16 ELISA at Smith College, Northampton, MA, USA. For the ELISA, blood spots were eluted from two 6-mm punches (see below). Each eluate (the product of two punches) was run in duplicate in the Ov16 ELISA to detect anti-Ov16 IgG4 antibodies.

#### 4.3.3 Ov16 IgG4 detection using ELISA

Blood spots were eluted if their quality allowed for two full 6-mm punches to be taken from the saturated area. These two punches were then transferred to a single well within a 96-well elution plate (Corning® 3788), with the sample ID recorded in an elution plate map. 100µL of DBS elution buffer (phosphate buffered saline (PBS) + 0.05 % Tween + 5 % bovine serum albumin (BSA)) was added to each well containing the two DBS punches making sure that the punches were completely submerged in the buffer, with no air bubbles surrounding them. Plates were covered and incubated overnight (12–24 h) in a refrigerator at 2–8 °C [18].

Immulon 2HB plates (Thermo Scientific, Waltham, MA, USA) were coated with Ov16 antigen. For coating, stock antigen at a concentration of 5 µg/mL was diluted 1:100 with PBS. Once plated, the coating solution was refrigerated at 4 °C overnight and subsequently removed via inversion of the plates. Plates were then blocked with 200µL/well of blocking solution (2.5 mL of foetal bovine serum (FBS), 25 mL of PBS-tween (PBST) and 22.5 mL of ddH_2_O) for 30 min in a 37 °C incubator. The wells were then washed three times with 300µL of PBST.

Control sera were subsequently prepared. The negative control consisted of 50µl of PBST+ 5 % FBS, with the positive control being a dilution panel of AbD_32 antibody (PATH, Seattle, WA, USA) (2000 ng/mL stock) diluted into PBST + 5 % FBS at concentrations: 20 ng/mL, 10 ng/mL, 5 ng/mL, 2.5 ng/mL, 1 ng/mL, 0.5 ng/mL, 0.25 ng/mL, 0.1 ng/mL. The remainder of the wells contained 50µL of the DBS eluates, with each eluate (collected from two DBS punches) run in duplicate. An eluted known positive DBS sample labelled ‘DBS Positive 250 ng/mL’ was also run in duplicate.

Once added into the designated wells, the plates were incubated for a further hour at 37 °C and washed three times with 300µL of PBST. Mouse anti-human IgG4 antibody (HP6025) (Hybridoma Reagent Laboratory, Baltimore, MD, USA) was added to each well at a ratio of 1:5000. The antibody was diluted in PBST + 5 % FBS and 50 µL was added to each well. The plates were further incubated for 1 hour at 37 °C and washed three additional times with 300µL of PBST.

50 µL of a goat anti-mouse secondary antibody conjugated to horseradish peroxidase (GαM-HRP) (Jackson Immuno, West Grove, PA, USA), diluted in PBST + 5 % FBS, was added to each well at a ratio of 1:10,000. The plates were further incubated for 1 hour at 37 °C and washed three additional times using 300µL of PBST.

3,3,5,5-Tetramethyl Benzidine (TMB) (Sigma, Mumbai, India) was equilibrated to room temperature. 100 µL of TMB was added per well and incubated at room temperature for a further 15 min. The enzymatic reaction was stopped by adding 50 µL of 1 N HCl to each well. The plates were placed in the plate reader (SpectraMax M2, Molecular Devices, San Jose, CA, USA), with the endpoint ELISA absorbance (optical density, OD) read at 450 nm.

The cut-off values were determined as the mean absorbance of the negative control wells plus three times the standard deviation. If the OD of the sample was above the cut-off, the sample was designated as a positive result.

Standard curves were generated for each plate by plotting known concentrations of the positive control (AbD_32) dilution series (described above). A 4-parameter logistic regression model was used to fit the standard curve,

(1) $$x=c\;{\left(\frac{a-d}{y-d}-1\right)}^{1/b}$$

where x is the inferred concentration, y is the measured OD, a and d are, respectively, the minimum and maximum responses, c represents the point of inflection and b the gradient of the curve at the inflection point. The standard curve was used to generate inferred concentrations from the OD readings of the positive samples. Samples identified to have OD readings inconsistent with the standard curve, and which could not be rectified by re-assaying, were deemed invalid (*n* = 22) and excluded, leaving 2990 valid samples for analysis [18].

#### 4.3.4 Data analysis

**Fig. 1:**
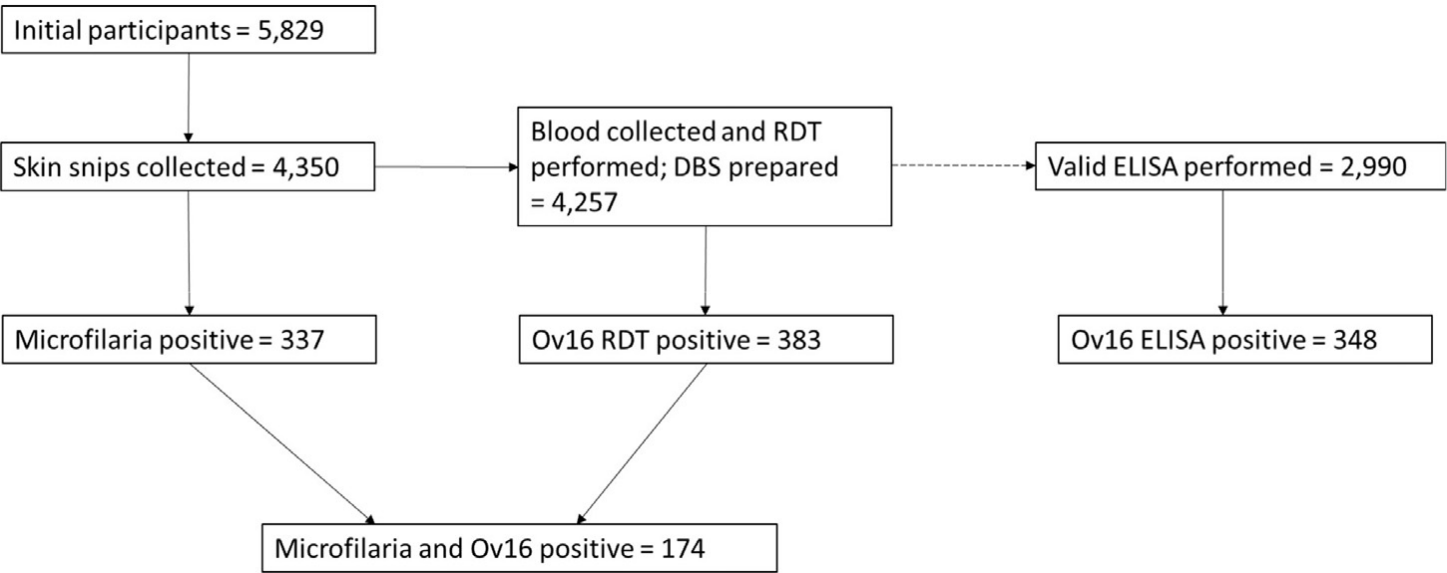
Participant testing flowchart. Of an initial 5829 participants, skin-snip samples were collected from 4350 (75 %) and blood samples from 4257 participants (98 % of those skin-snip tested; solid horizontal arrow). All 4257 were tested using Ov16 RDT with whole blood and dried blood spots (DBS) were prepared. After assessing DBS quality, 2990 (70 %) had a valid test using Ov16 ELISA (dashed horizontal arrow). A total of 337 participants (8 %) were positive for skin-snip microscopy, 383 (9 %) were seropositive for Ov16 RDT and 348 (12 %) for Ov16 ELISA. Of the 4257 participants, 174 individuals tested positive for both skin- snip microscopy and RDT. Of the 2990 DBS samples which underwent valid testing with Ov16 ELISA, 156 were positive for both skin-snip and ELISA, and 192 were negative for skin-snip but positive for ELISA (for a total of 348 ELISA-positive). When calculated against skin-snip microscopy, the sensitivity and specificity of the Ov16 RDT were, respectively, 52 % and 95 %. The sensitivity and specificity of the Ov16 ELISA were, respectively, 59 % and 93 %.

**Fig. 2:**
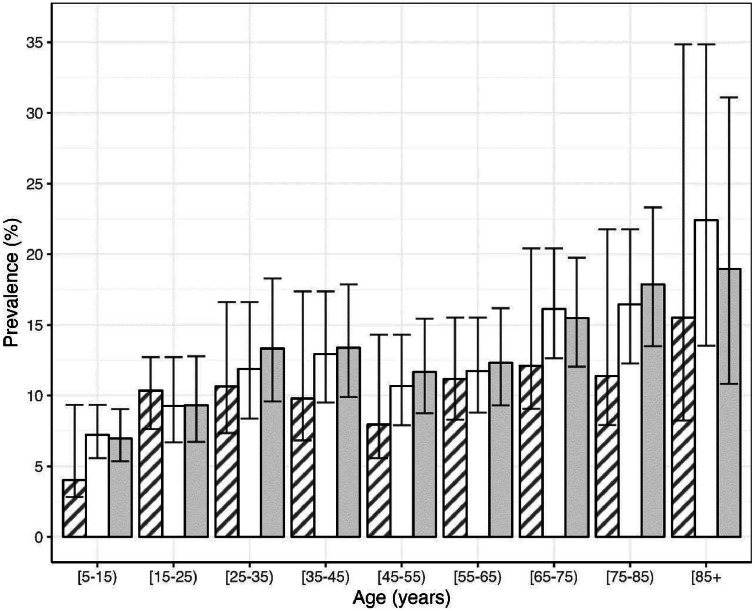
Age-profiles of prevalence according to diagnostic in the onchocerciasis delineation mapping dataset from Gabon, 2015. Samples were collected in 67 communities in 14 districts from consenting participants aged ≥ 5 years. *Onchocerca volvulus* microfilarial prevalence measured using skin-snip microscopy (*n* = 4350; grey- dashed bars), IgG4 antibody prevalence to the *O. volvulus* Ov16 antigen measured using rapid diagnostic test (Ov16 RDT; *n* = 4257; white-bars) and IgG4 antibody prevalence to the *O. volvulus* Ov16 antigen measured by ELISA (*n* = 2990; grey-bars). Error bars indicate Wilson score 95 % confidence intervals [13].

**Fig. 3:**
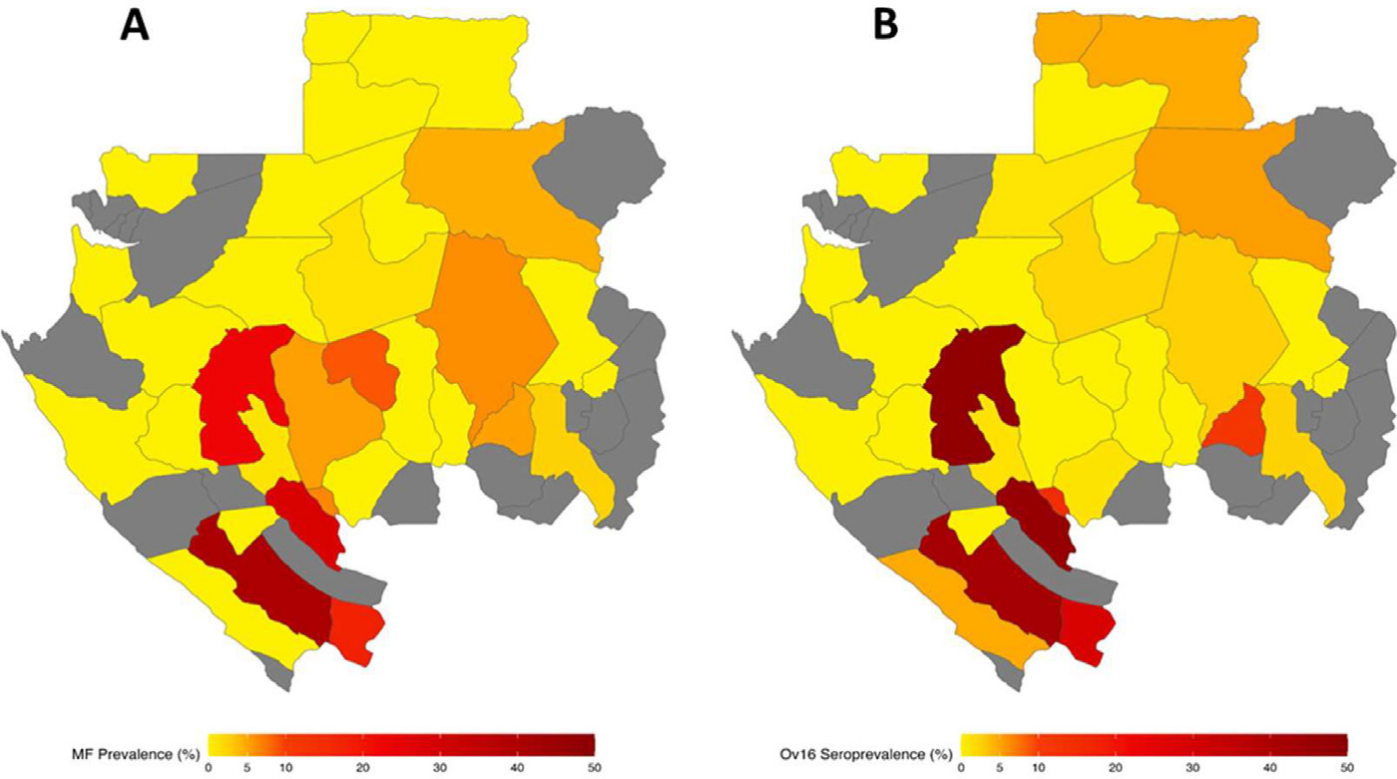
Geographical comparison of *Onchocerca volvulus* microfilarial prevalence and Ov16 seroprevalence in Gabon, 2015*.* Of 51 implementation units (IUs), sampling took place in 31, with 20 (grey-shaded) not sampled. Yellow-shaded IUs show the lowest recorded prevalence and darkest red-shaded IUs show the highest prevalence. Panel A: microfilarial prevalence measured by skin-snip microscopy; Panel B: Ov16 seroprevalence measured by rapid diagnostic test (Ov16 RDT) as described in the text. Samples were collected prior to implementation of ivermectin treatment. Districts where sampling was undertaken were located within IUs as mapped in the Expanded Special Project for Elimination of Neglected Tropical Diseases (ESPEN) portal for Gabon [14].

Raw data were collated using MS Excel, being cleaned and analysed using R, version 4.33 [19]. Ninety-five percent confidence intervals (95 % CI) were calculated using the Wilson score method [13]. Analysed data were visualised using R packages: ggplot2, patchwork (plotting and arrangement) and sf (mapping) using shape files for implementation units of Gabon, sourced from the ESPEN portal [14]. Fig. 1,Fig. 2,Fig. 3,Table 1

**Table 1:** Order and content of dataset [1].

Excel Sheet	Variable	Variable Description
		
**1: “Gabon Data”**	Village Code	Identification (ID) number assigned to each village from which participants were recruited
	Family No.	Unique identifier for individuals in the same family unit
	Order No.	Numerical order in which individuals were sampled
	Individual ID	Unique ID for each participant
	Sex	1 = Male; 2 = Female
	Age	Participant age in years at time of sample collection
	Exam Status	Whether an individual had undergone examination for skin microfilariae (1 = Yes; 2 =No; 9 = Unknown)
	Resident Duration	How long (in years) a participant had lived in their village up to sample collection
	Ov16 Result	A participant's Ov16 status as determined by Ov16 RDT. 1 = Positive; 2 = Negative
	Oncho MF Snip Left	Enumeration of microfilariae emergent from the participant's left iliac crest during skin snip microscopy
	Oncho MF Snip Right	Enumeration of microfilariae emergent from the participant's right iliac crest during skin snip microscopy
	Oncho MF Mean by Snip	Mean number of microfilariae across both iliac crests from a participant
	Survey Date	Date participant was surveyed
	Oncho mf Status	Status of a participant's skin-snip microscopy result. Positif = Positive; Negatif = Negative
	Oncho Ov16 Status	Status of a participant's Ov16 RDT result. Positif = Positive; Negatif = Negative
	Region	National subdivision of Gabon within which participant was sampled
	District Name	Regional subdivision of Gabon within which participant was sampled
		
**2: “Gabon Data by Village”**	Village	Named location in which participants were sampled
	MF Negative	Count of individuals whose skin-snip microscopy status was negative
	MF Positive	Count of individuals whose skin-snip microscopy status was positive
	Total	Sum of individuals sampled via skin snip-microscopy
	% Pos MF	Percentage of skin snip positive individuals out of the total sampled
	Class. (MF only)	Determined level of endemicity based on% Pos MF; ≤ 40 % = hypoendemic, >40 % = *meso*/hyperendemic
	Ov16 Neg| MF pos	Individuals who returned a negative Ov16 RDT result with a positive result from skin snip microscopy
	Ov16 Pos| MF Pos	Individuals who returned a positive Ov16 RDT result with a positive result from skin snip microscopy
	Ov16 RDT Sensitivity	The percentage of Ov16 Pos| MF Pos individuals out of the number of MF positive individuals giving sensitivity of Ov16 RDT when measured against skin snip microscopy
	Ov16 Neg| MF neg	Individuals who returned a negative Ov16 RDT result with a negative result from skin snip microscopy
	Ov16 Pos| MF neg	Individuals who returned a positive Ov16 RDT result with a negative result from skin snip microscopy
	Ov16 RDT Positive	Number of individuals with a positive Ov16 RDT
	Ov16 Neg Total	Number of individuals with a negative Ov16 RDT
	Ov16 RDT Specificity	The percentage of Ov16 Neg| MF neg individuals out of the number of MF negative individuals giving specificity of Ov16 RDT when measured against skin snip microscopy
	% pos Ov16 RDT	Percentage of individuals Ov16 RDT Positive out of the Total
	Class (Ov16 only)	Determined level of endemicity based on% pos Ov16 RDT; ≤ 40 % = hypoendemic, >40 % = *meso*/hyperendemic
	% pos OV16 or MF	Percentage of individuals out of the Total with either a positive snip microscopy result or positive Ov16 RDT
	Class. (Ov16 or MF)	Determined level of endemicity based on% pos Ov16 or MF; ≤ 40 % = hypoendemic, >40 % = *meso*/hyperendemic
	Ov16 ELISA Results	Percentage of individuals with a positive Ov16 ELISA
		
**3: “Ov16 Gabon ELISA”**	Individual ID	Unique identifier for each participant
	Ov16 status - RDT	Participant's Ov16 status as determined by RDT: 1 = Positive; 0 = Negative
	mf status	Result of the participant's skin-snip microscopy test: 1 = mf positive; 0 = mf negative
	Sample ID	Unique identifier for a participant's eluted dried blood spot sample (to be analysed via Ov16 ELISA)
	Inferred Conc.	Inferred concentration of IgG4 to Ov16 from optical density of positive samples (0 = negative sample)
	Region	National subdivision of Gabon within which participant was sampled
	District Name	Regional subdivision of Gabon within which participant was sampled
	Village Name	Named location in which participants were sampled
	Ov16 ELISA Plate #	Each village corresponded to a unique Ov16 ELISA plate number
	Sample ID/ Ind ID mismatch	Check to ensure sample and individual ID numbers matched. 0 = match; 1 = mismatch
	Ov16 ELISA Result	Status of individual's Ov16 ELISA test. Invalid = could not be determined; positive = above cut-off; negative = below cut-off
	Ov16 ELISA binary result	Ov16 ELISA Result expressed numerically. Invalid/negative = 0; positive = 1
	Age	Participant age in years at time of sample collection
	Age Group	Categorisation of participant age

## Limitations

Ov16 ELISAs were only conducted on DBS samples deemed to be of sufficient quality. As such, the sample size for Ov16 ELISA is smaller compared to the other two diagnostics. Caution must therefore be applied when interpreting the diagnostic performance of the Ov16 ELISA. Further, the Ov16 ELISA data do not include the sex of the participants.

## Ethics Statement

All surveys and sample collections were conducted after receiving informed consent from the participants. If the participant was aged 15 years or younger, the permission of a legal parent or guardian was obtained. Ethical approval was granted prior to the commencement of the study from the Ministry of Health and Social Affairs of the Republic of Gabon, Reference: APOC/EVE/030/15/HZ. In addition, we confirm that all authors have abided by the ethical requirements for publication in *Data in Brief* and that our work did not involve the use of animal experiments or the collection of data from social media platforms.

## CRediT authorship contribution statement

**Julienne Atsame:** Conceptualization, Project administration, Resources. **Jacob N. Stapley:** Formal analysis, Data curation, Visualization, Writing – original draft. **Aditya Ramani:** Formal analysis, Data curation, Visualization. **Romain Mourou:** Methodology, Supervision. **Ella Ntsame:** Methodology. **Eya Efame:** Data curation. **Ollomo-Nziengui Angue:** Investigation. **Jean-Luc Obiang:** Investigation. **Nils Pilotte:** Investigation, Methodology, Data curation, Resources, Writing – review & editing. **Katherine Gass:** Conceptualization, Project administration, Writing – review & editing. **Maria-Gloria Basáñez:** Supervision, Visualization, Writing – original draft, Writing – review & editing.

## Data Availability

Dataset of the performance of Ov16 RDTs versus skin snip microscopy and Ov16 ELISA, Gabon, 2015 (Original data) (Mendeley Data).

## References

[1] J.N. Stapley. Dataset of the performance of Ov16 RDTs versus skin snip microscopy and Ov16 ELISA, Gabon, 2015, V2. Mendeley Data (2024), 10.17632/vtvmrzs9ch.2. Available: https://data.mendeley.com/datasets/vtvmrzs9ch/2.

[2] World Health Organization. A handbook for onchocerciasis elimination mapping (OEM). In Press. (2024).

[3] Dieye Y., Storey H.L., Barrett K.L., Gerth-Guyette E., Di Giorgio L., Golden A., Faulx D., Kalnoky M., Ndiaye M.K.N., Sy N., Mané M., Faye B., Sarr M., Dioukhane E.M., Peck R.B., Guinot P., de Los Santos T. (2017). Feasibility of utilizing the SD BIOLINE Onchocerciasis IgG4 rapid test in onchocerciasis surveillance in Senegal. PLoS Negl. Trop. Dis..

[4] Otabil K.B., Basáñez M.G., Ameyaa E., Oppong M., Mensah P., Gyasi-Ampofo R., Bart-Plange E.J., Nti Babae T., Datsa L., Boakye A.A., Yeboah M.T. (2024). Usability, acceptability and cost of the SD BIOLINE Ov16 rapid diagnostic test for onchocerciasis surveillance in endemic communities in the middle belt of Ghana. medRxiv.

[5] Hamley J.I.D., Milton P., Walker M., Basáñez M.G. (2019). Modelling exposure heterogeneity and density dependence in onchocerciasis using a novel individual-based transmission model, EPIONCHO-IBM: implications for elimination and data needs. PLoS Negl. Trop. Dis..

[6] Hamley J.I.D., Walker M., Coffeng L.E., Milton P., de Vlas S.J., Stolk W.A., Basáñez M.G. (2020). Structural uncertainty in onchocerciasis transmission models influences the estimation of elimination thresholds and selection of age groups for seromonitoring. J. Infect. Dis..

[7] Lont Y.L., Coffeng L.E., de Vlas S.J., Golden A., de Los Santos T., Domingo G.J., Stolk W.A. (2017). Modelling anti-Ov16 IgG4 antibody prevalence as an indicator for evaluation and decision making in onchocerciasis elimination programmes. PLoS Negl. Trop. Dis..

[8] Coffeng L.E., Stolk W.A., Golden A., de Los Santos T., Domingo G.J., de Vlas S.J. (2019). Predictive value of Ov16 antibody prevalence in different subpopulations for elimination of African onchocerciasis. Am. J. Epidemiol..

[9] World Health Organization & African Programme for Onchocerciasis Control. Conceptual and Operational Framework of Onchocerciasis Elimination with Ivermectin Treatment (2010). Available: https://iris.who.int/handle/10665/275466.

[10] Biamonte M.A., Cantey P.T., Coulibaly Y.I., Gass K.M., Hamill L.C., Hanna C., Lammie P.J., Kamgno J., Nutman T.B., Oguttu D.W., Sankara D.P., Stolk W.A., Unnasch T.R. (2022). Onchocerciasis: target product profiles of in vitro diagnostics to support onchocerciasis elimination mapping and mass drug administration stopping decisions. PLoS Negl. Trop. Dis..

[11] African Programme for Onchocerciasis Control. Report of the Consultative Meetings on Strategic Options and Alternative Treatment Strategies for Accelerating Onchocerciasis Elimination in Africa (2015). Available: https://tdr.who.int/publications/m/item/report-of-the-consultative-meetings-on-strategic-options-and-alternative-treatment-strategies-for-accelerating-onchocerciasis-elimination-in-africa.

[12] Coalition for Operational Research on Neglected Tropical Diseases. Multi-country comparison of diagnostics tools for Onchocerca volvulus, Wuchereria bancrofti and Loa loa (2015). Available: https://www.cor-ntd.org/research-outcomes/studies/multi-country-comparison-diagnostics-tools-onchocerca-volvulus-wuchereria

[13] Brown L.D., Cai T.T., DasGupta A. (2001). Interval estimation for a binomial proportion. Statist. Sci..

[14] Expanded Special Project for the Elimination of Neglected Tropical Diseases. National summary statistics for Gabon (2024). Available: https://espen.afro.who.int/countries/gabon.

[15] Zouré H.G.M., Noma M., Tekle A.H., Amazigo U.V., Diggle P.J., Giorgi E., Remme J.H.F. (2014). The geographic distribution of onchocerciasis in the 20 participating countries of the African Programme for Onchocerciasis Control: (2) pre-control endemicity levels and estimated number infected. Parasit. Vectors.

[16] Zouré H.G.M., Wanji S., Noma M., Amazigo U.V., Diggle P.J., Tekle A.H., Remme J.H.F. (2011). The geographic distribution of Loa loa in Africa: results of large-scale implementation of the Rapid Assessment Procedure for Loiasis (RAPLOA). PLoS Negl. Trop. Dis..

[17] Moreau J.P., Prost A., Prod'hon J. (1978). Essai de normalisation de la méthodologie des enquêtes clinco-parasitologiques sur l'onchocercose en Afrique de l'Ouest [An attempt to normalize the methodology of clinico parasitologic surveys of onchocerciasis in West-Africa (author's transl)]. Med. Trop..

[18] Coalition for Operational Research on Neglected Tropical Diseases. A protocol and annex for a multi-country comparison of diagnostics tools for Onchocerca volvulus, Wuchereria bancrofti and Loa loa (2015). Available: https://www.cor-ntd.org/research-outcomes/studies/multi-country-comparison-diagnostics-tools-onchocerca-volvulus-wuchereria.

[19] R Core Team. R: a language and environment for statistical computing. R. version 4.3.3 (2023). Available: https://www.r-project.org/.

